# Commentary: A longitudinal exploration of the effects of the COVID‐19 lockdowns for adolescents both with and without neurodevelopmental disorders – a reflection on Houghton et al. (2022)

**DOI:** 10.1111/jcpp.13661

**Published:** 2022-06-30

**Authors:** Charlotte Field

**Affiliations:** ^1^ School of Psychology and Computer Science University of Central Lancashire Lancashire UK

## Abstract

The lockdowns during the COVID‐19 pandemic had and continue to have severe and wide‐ranging effects worldwide on mental health and loneliness. In this commentary, I summarise Houghton et al. (2022) who explored these effects longitudinally in adolescents in Western Australia, with and without a Neurodevelopmental Disorder (NDD), considering the strengths and weaknesses of the article and its importance to the field. Adolescents with NDD, who already had a high baseline rate of loneliness and mental health difficulties, did not find that this increased during COVID‐19 lockdowns. However, adolescents without NDD, who began with a much lower baseline rate, found that this was elevated. There was variability in terms of different types of NDD, with adolescents who had ADHD reporting some positive effects of the COVID‐19 lockdowns. These findings highlight the importance of support for adolescents both with NDD and those without as the world emerges out of the pandemic.

Adolescence is a key period of development, with multiple changes and adjustments in terms of biological, cognitive, social and emotional growth making this age a critical period for communal interaction and social sensitivity (Blakemore & Mills, 2014). The worldwide unprecedented circumstances of the COVID‐19 pandemic, including ‘stay‐at‐home’ (‘lockdown’) orders, school closures and social distancing may have led to an increase in depression, mental health difficulties and loneliness for adolescents (Ellis, Dumas, & Forbes, 2020). Furthermore, adolescents with Neurodevelopmental Disorders (NDDs) might be affected differently during lockdown from their peers. Given the symptoms frequently seen in NDD, there might be both negative (e.g. struggling to adapt to changes in routines caused by restrictions) and/or positive (e.g. relief of fewer social activities) aspects of the COVID‐19 lockdowns. Houghton et al. (2022) investigate these factors in a longitudinal study of the impact of COVID‐19 restrictions in adolescents with and without NDD (both *N* = 238) in Western Australia.

Within the population of adolescents without NDD, given the increased vulnerability for mental health difficulties and loneliness during this developmental time period and the importance of a strong friendship group to help support adolescents, it might be expected for the COVID‐19 pandemic lockdown restrictions to have had a secondary negative effect on many adolescents due to being unable to see friends and attend school (e.g. Ellis et al., 2020). Friendship groups and school structure might help with loneliness during adolescence, acting as a buffer to prevent mental health difficulties. However, it is also worth noting the high technology, digital and social media usage among adolescents which might have meant that they were generally able to keep in touch with friends and attend social events online during the pandemic (Cauberghe, Van Wesenbeeck, De Jans, Hudders, & Ponnet, 2021). Furthermore, some adolescents (particularly those who are more introverted) may have enjoyed spending more time at home.

One currently somewhat neglected group of adolescents in terms of the impact of COVID‐19 restrictions is adolescents with various forms of NDD. These individuals might have been more susceptible to negative effects of lockdown than their peers due to unpredictable and changing routines, the loss of the structure that the school day provides and uncertainty in terms of lockdown restrictions. Past research (e.g. Breaux et al., 2021; Kawaoka et al., 2021) has found adverse effects of lockdown on adolescents with NDD. However, individuals with NDD with social difficulties might experience more time spent alone and generally socialising less than others, which might mean being better accustomed to ‘social isolation’ than peers without NDD prior to the pandemic. Furthermore, those with NDD often experience difficulties at school and other social situations, meaning that enforced restrictions may have provided relief from ever changing and confusing past obligations (e.g. Cassidy et al., 2020; Dekker et al., 2022).

Understandably, given the sudden and recent onset of COVID‐19, many studies within this field so far have been cross sectional, occurring during one time point during COVID and some research involving individuals with NDD lacks a control group. Two years on from the beginning of the pandemic, as it hopefully begins to stabilise and the world heads more towards ‘normality’, longitudinal studies investigating the effect of the lockdowns at various different stages of lockdown and restrictions will start to emerge. Houghton et al. (2022) begin to fill the current gap in longitudinal research, by specifically exploring implications for mental health and loneliness across two separate populations of adolescents; those with and without NDD longitudinally during the pandemic, over two‐and‐a‐half years. This involved before (November 2018, April 2019, March 2020) and after (July/August 2020) the schools closed. There is an impressive sample size, with the participants with NDD matched to the control group on age and sex. Adolescents with five different types of NDDs are considered; Autism Spectrum Disorder (ASD; *N* = 23), Attention Deficit Hyperactivity Disorder (ADHD; *N* = 55), Specific Learning Disorders (SLD; *N* = 119), two or more diagnoses (*N* = 25) and NDD of unknown aetiology (*N* = 16). Therefore, this research fills various important gaps left by past studies within the field, investigating the effects of the pandemic longitudinally, with adolescents and with varying different types of NDD compared with a control group.

Across four waves of data collection, adolescents with NDD self‐reported elevated levels of poor mental health compared with their peers, via a variety of scales measuring loneliness, depression, mental wellbeing and strengths and difficulties in socialisation. However, there was little change over time in terms of mental health for adolescents with NDD. In contrast adolescents without NDD, who originally had low symptoms of depression, experienced a significant increase of various mental health symptoms over time during lockdown. Therefore, the unpredictability of the COVID‐19 lockdowns was not associated with poor mental health in adolescents with NDD. However, it was linked with a decline in mental health for those without. Potential explanations for these findings concern the social difficulties often experienced by adolescents with NDD. They may have been more used to being alone and awarded some relief from stressful in‐person social activities via lockdown, using social media to stay in touch with friends. However, those without NDD suddenly and unexpectedly experienced social isolation and were not able to engage in previously enjoyed social experiences hence had a decline in mental health. These explanations are admittedly post hoc and it is also important to consider individual differences here; not everyone with NDD has social difficulties and some of the sample of those without NDD fared better from lockdown than others. Importantly, some of the sample will have been affected by COVID‐19 during the lockdown more than others, for example, via own illness or illness/bereavement of someone close to them, loss of job of family member, more stress within the family home, etc. These individual differences were not explored within the paper, but would be an interesting area for future research.

Nevertheless, the work of Houghton et al. (2022) is extremely important given the lack of past longitudinal research in this area and the wide‐ranging potential theoretical and applied implications of these results. There is thorough consideration about the effect that the lockdowns have had on adolescents both with and without NDD. Interestingly, the findings of the paper suggest that the adolescent population *without NDD* might have been particularly vulnerable to the negative effects of lockdown. Thus, these individuals may benefit from mental health interventions in the aftermath of the COVID‐19 pandemic, as they experienced significant declines in positive wellbeing and increases in depressive‐type symptoms. As adolescents begin to socialise again and ‘re‐learn’ the pre‐pandemic social experiences, routines and school structure, it is very important to consider the mental health and loneliness these participants tended to experience during the restrictions.

Unlike their typically developing peers, adolescents with NDD had a concerningly high baseline rate of poor mental health and loneliness. However, they did not show negative mental health outcomes post lockdown/school closure. The increase in online activities, less pressure to attempt to conform to societal expectations and becoming more used to being alone might all be seen as an advantage for those with NND during lockdown but a disadvantage for those without. As individuals with NDD might be used to being lonelier in general (as demonstrated by their higher loneliness than the control group at the beginning) lockdown might not have made as much difference as compared with the control group who suddenly, dramatically and unexpectedly found themselves isolated. Indeed, the adolescents with NDD might have felt more ‘connected’ with others than previously as everyone was now staying at home with social interaction difficulties. This could explain why pre‐COVID there were higher levels of isolation and lower positive attitudes to being alone in the adolescents who had NDD.

Crucially however, there were some differences in different subgroups of neurodevelopmental disorders. For adolescents with ASD and SLD, the COVID‐19 lockdowns were not associated with adverse impairment. For adolescents with ADHD there was *higher* positive mental wellbeing and decreases in externalising symptoms in between pre‐COVID and post schools re‐opening. This is in contrast to past research finding adverse effects of lockdown for adolescents with ADHD (e.g. Breaux et al., 2021). As the authors themselves highlight, future research should see if these effects are replicated across different samples and cross‐culturally.

The Houghton et al. (2022) paper makes an important contribution to the study of mental health, NDD and adolescents. From an educational and clinical perspective, interventions should target help for adolescents both with and without NDD as the world begins to ‘emerge’ from COVID‐19. The very high rates of mental health problems and loneliness even at baseline for those with NDD are concerning and future research and interventions should help to alleviate this. Nevertheless, unlike their peers, this group did not experience increased mental health and loneliness difficulties through lockdown.

It would be interesting to tease apart the qualitative reasons for differences between how adolescents with and without NDD perceived lockdown and if experiences during the lockdown could be simulated to assist those with NDD in future. For example, an increase in digital and technical activity and different routines that those with NDD might have experienced during lockdown, if helpful, might be applied to some social activities. The in‐person school experience is of vital importance, as this study highlights and use of online/digital technology is no substitute for this. However, more hybrid models of working and socialising, such as making some events available online as well as in person might help facilitate engagement for some individuals with NDD (as well as other individuals who might for various reasons have difficulties attending in person or prefer to attend online). As we progress from the pandemic, this study paves the way for future longitudinal and cross‐cultural research about the secondary effects of COVID‐19 restrictions on adolescents both with and without NDD.
